# Advancements in Biomedical Sensors for Early Detection of Failure in Hip and Knee Implants: Scoping Review on Potential Sensors for Implant Integration

**DOI:** 10.1007/s10439-025-03780-5

**Published:** 2025-07-02

**Authors:** P. H. Helene Noordhuis, Paul C. Jutte, Ajay G. P. Kottapalli, Claudine J. C. Lamoth, C. C. Charissa Roossien

**Affiliations:** 1 Department of Human Movement Science, University Medical Center Groningen, University of Groningen, Hanzeplein 1, 9713 GZ Groningen, The Netherlands; 2 Department of Orthopaedics, University Medical Center Groningen, University of Groningen, Hanzeplein 1, 9713 GZ Groningen, The Netherlands; 3 Department of Bio-inspired MEMS and Biomedical Devices, Faculty of Science and Engineering, Engineering and Technology Institute Groningen (ENTEG), University of Groningen, Nijenborgh 4, 9747 AG Groningen, The Netherlands

**Keywords:** Biomedical measurements, Failure analysis, Hip, Implantable biomedical devices, Knee, Prosthetics, Sensors

## Abstract

**Purpose:**

Despite significant advancements in hip and knee joint implant technology, 6.4% of implants fail within the first ten years due to aseptic loosening, instability, and/or infection. Implants equipped with sensors show promise in early failure detection, enabling early and reduced intervention. This scoping review aims to provide an overview of biomedical sensors and their potential for integration into hip- and knee implants.

**Methods:**

A comprehensive search of databases PubMed and Embase was performed. Inclusion criteria were sensors to detect failure causes infection, inflammation, loosening or wear; developed for biomedical applications; ex vivo, in vivo and/or in vitro studies. The sensors were analysed based on criteria per sensor characteristics (e.g. accuracy, durability, response time) relevant for implant integration.

**Results:**

49 articles were included presenting 52 sensors: 24 pressure and force, 6 strain, 15 acidity, 4 temperature, and 3 bacterial detection (3 dual sensing elements). Among these, three sensors were specifically designed for hip- and knee implants. The remaining 46 were developed for other biomedical applications. Our analysis identified two strain and seven acidity sensors that met the criteria for detecting hip- and knee implant failure. Two bacteria sensors showed potential for short-term use post-implantation, aligning with the critical period for periprosthetic infection, but the reporting frequency was too low to draw proper conclusions. No wear (particle) sensor was found.

**Conclusion:**

We found a significant gap in sensors that can detect wear particles. Future work on continuous implant monitoring should focus on reducing risk and the enhancement of sensor durability and longevity.

## Introduction

Worldwide the number of joint replacements is increasing rapidly [1]. Patients suffering from degenerative joint diseases such as osteoarthritis (OA) require surgical joint replacement to alleviate pain and restore function [2]. In 2020, 7.6% of the global population suffered from OA. Over the next 30 years an increase of more than 70% is estimated for hip and knee OA and more than 95% for other types of OA [3]. In the Netherlands, total hip arthroplasty (THA) is the most commonly performed joint implant surgery, closely followed by total knee arthroplasty [4]. Despite significant advancements in hip joint implant technology, 6.4% of implants fail within the first ten years due to aseptic loosening, instability, and/or infection [5, 6]. Over time, implants tend to fail more frequently because of wear [7, 8]. Therefore, the average lifespan of a hip implants ranges from 10 to 20 years [9] and for knee implants this is even shorter, about 5.9 years [10].

When implants fail, revision surgery becomes necessary. Revision surgery often bring complications, higher morbidity rates, and increased healthcare costs [2]. Patients can only undergo a limited number of revision surgeries since successively revised implants typically have less than half the lifespan of the previous [11]. Patients who receive a hip- or knee implant at a younger age have the lowest ten-year survival rate due to their active lifestyle and risk factors associated with why they received the implant [2, 12]. For patients between ages 46 and 50, the risk of needing hip implant revision surgery is 27.6%. [6]. Consequently, THA in patients under 50 is postponed for as long as possible, while these patients often endure chronic pain and risk losing their social and economic independence [13]. Hip or knee implant failure remains a significant challenge in orthopedic surgery, with the most common causes being infection (affecting 22% of knee and 18% of hip implants), aseptic loosening (35% for hips, 18% for knees), and wear (3.7% for hips, 1.6% for knees) [14]. Loosening of an orthopaedic implant can gradually degrade the surrounding bone, reducing the bone stock necessary for stable fixation of a revision implant and thereby lowering the likelihood of successful revision surgery [15]. Early detection of failure modes—such as loosening, wear, infection or inflammation—is critical. Early detection can delay or prevent the need for revision surgery while improving the probability of successful treatment for acute periprosthetic infection. This can be achieved by either preserving the original implant or enabling a less invasive, less expensive, and lower-risk revision procedure [16, 17]. Determining and detecting the onset of a knee- or hip implant failure, as well as the precise interaction between the implant and surrounding tissue, is challenging [15]. Current diagnostic methods rely on a combination of patient-reported pain, clinical physical examinations, imaging techniques, and laboratory analysis of blood, tissue biopsies, and synovial fluid [18, 19]. These techniques primarily focus on diagnosing symptoms that appear after implant failure has occurred, leading to delayed detection and potential misdiagnosis [20]. Earlier detection of hip- and knee implant failure could both postpone the need for revision surgery and allow for better surgical planning when revision becomes necessary [7, 8, 21].

Instrumented hip and knee implants equipped with sensing technology could monitor characteristics associated with loosening, wear, infection or inflammation that led to implant failure. While an integrated sensor system specifically developed for hip- and knee implants does not yet exist, sensors developed for other biomedical applications might have potential for integration in an instrumented hip- and knee implant. These sensors should be able to monitor key failure indicators: biochemical changes (e.g., increased temperature, increased temperature from friction or inflammation, synovial fluid viscosity, acidity, lactate dehydrogenase), and mechanical changes (e.g., position, force, pressure, motion). Wear-induced friction generates particulate debris of the articulating areas, triggering inflammation and, in some cases, infection [22]. These biological responses elevate pro-inflammatory cytokines and recruit immune and bone-associated cells such as macrophages, monocytes, fibroblasts, osteoblasts, osteoclasts, and mesenchymal stem cells in the joint environment [23]. This process raises levels of red blood cells, lactate dehydrogenase, and proteins while lowering glucose levels in the synovial fluid. The fluid may also contain crystals and microorganisms. These changes affect the fluid's colour, clarity, and viscosity, as well as increase its volume —further signalling pathological changes [24]. The resulting rise in joint pressure and temperature contributes to implant loosening and additional particle generation, particularly via micromotions of the implant [21, 24, 25]. While infections typically occur shortly after implantation, inflammation and loosening can manifest years later [26]. Additional requirements for sensors concern the sensor's size, if integrated inside the implant, the sensor must fit within the implant's dimensions. If placed near the implant, the implant's size can serve as a size guideline and the sensor should not interfere with the implant function. Potential sensors should be compatible with the physical and biological environment of a hip- or knee implant over an extended period. This excludes materials with limited longevity, such as biodegradable, bioresorbable, or single-use components. For continuous monitoring, detecting trends over time, periodically measured, is more relevant than high-frequency measurements, as most hip- and knee implant failures develop gradually, such as due to wear and infection [26, 27]. For mechanical sensors, the forces exerted on a hip- and knee3 implant can reach up to nearly 500% of body weight [28]. Previous measurements in knee implants using strain sensors have shown a measuring range of 0 to 900 or 1500 N [29, 30]. With a hip implant contact area of 300mm^2^ [31], these forces result in pressures of 3–5 MPa during daily activities. Knee implants have a larger contact area of ~ 650 mm^2^ [32], resulting in lower pressures of 1.4–2.3 MPa. Any integrated sensors must withstand these forces and have an appropriate measuring range. Additionally, the distance from skin to the bone tip where a hip implant is placed averages 76 mm [33], while in the knee this distance is 10.2 mm from skin to bone over the medial epicondyle [34]. Therefore, the sensor's maximum measuring depth must exceed these distances.

This scoping review aims to provide a comprehensive overview of sensors with biomedical applications and their potential for integration into hip- and knee implants. Our review covers sensors currently used in medical practice or under research (in vitro or in vivo tested) for biomedical applications sensing one or more indicators that might indicate hip- and knee implant failure. The sensors of interest should have undergone ex vivo, in vivo and/or in vitro studies, and have the potential to meet the above criteria. We assess the reported sensor characteristics (e.g., measurement parameter, size, sensitivity, resolution, response time, measuring speed, measurement range, measuring depth, linearity, accuracy, precision, drift, and hysteresis) to investigate/map their potential for integration into sensorized implants.

## Methods

### Information Sources and Eligibility Criteria

The information for this scoping review was gathered via a comprehensive search of the two databases PubMed and Embase. For both databases a search query was built using Medical Subject Headings (MeSH terms) and free terms e.g. pressure sensor, strain gage which are not included in the MeSH terms. These terms were based on biosensing techniques, types of sensors or factors to be measured, sensor characteristics, and exclusions (see Appendix 7.1, 7.2). The search for both databases was executed on April 15, 2024, with a filter to select only articles published from 2013 to 2024. This review followed the PRISMA guidelines for systematic reviews [35].

Studies were included if they described an in vivo or in vitro study examining a sensing technique used to measure one or more factors that might indicate hip- and knee implant failure (a change in pressure, friction, position, motion, wear, lactate dehydrogenase, staphylococcus (various species), C-reactive protein, procalcitonin, viscosity of synovial fluid, white blood cell count, temperature, and acidity). Studies were excluded if they (a) were not published in the last ten years, (b) not intended for in vivo implantation or continuous contact with bodily fluids/tissues; (c) focused on biodegradable or bioabsorbable materials; (d) lacked the ability to measure at least weekly over an extended period (e.g., one-time-use products); (e) did not include a laboratory study; or (f) focused on measuring factors not mentioned in the inclusion criteria.

### Study Selection and Risk of Bias Assessment

The reference managers Rayyan [36] and EndNote 20 [37] were used. After retrieval of the studies from the databases, the duplicate results were taken out using EndNote 20 [38]. The remaining articles underwent screening based on title and abstract by two independent reviewers (first search: PHN and SEK, second search: PHN and CJCL) using Rayyan, applying the inclusion criteria. A meeting resolved any disagreements between reviewers. An expert in the field performed a post-check on several random articles'abstracts and titles to ensure reliable screening. Subsequently, the remaining articles were screened based on full text by the same pairs of independent reviewers, using identical inclusion criteria. Another meeting resolved any disagreements. Reasons for exclusion were: (1) the sensor mentioned in the article is not meant for in vivo use; (2) the sensor is designed for one-time use, not continuous measurement; (3) data extraction from the sensor requires imaging techniques; (4) the sensor is not the main topic, or no sensor is mentioned; (5) the article only discusses simulations, not in vitro or in vivo tests; (6) the article is not an in vitro or in vivo study. The risk of bias of each article was determined by extracting reporting in two domains: (1) conflict of interest and (2) the percentage of sensor characteristics reported. This was done using the risk of bias visualization tool Robvis and its instructions [39]. The tool was adjusted to fit this study. For each domain four levels of bias can be assigned: (a) low, (b) some concerns, (c) high, and (d) no information. For domain one these levels translate respectively to: (a) reported, none of the authors have a conflict of interest, (b) reported, one of the authors has a conflict of interest, (c) reported, two or more of the authors have a conflict of interest, and (d) not reported. For domain two these levels translate to: (a) more than 40 percent of sensor characteristics is reported, (b) between 20 and 40 percent of sensor characteristics is reported, (c) less than 20 percent of sensor characteristics is reported. These percentages have been based on the highest reporting frequency over all studies of 7 out of 13 sensor characteristics (54%).

### Data Extraction and Analysis

The data extracted from each article encompassed the following topics: (a) General publication details, including the authors and the year of publication; (b) study type: whether in vitro, in vivo, or ex vivo studies were performed; (c) sensor application: the environment for which the sensor was designed, the parameters measured with the sensor; (d) sensor characteristics: measuring range, sensitivity, resolution, accuracy, precision, durability, the size of the sensor, and the size of the effective sensing area on the sensor, response time, measuring speed, measuring depth, linearity,, drift, and hysteresis (see table 1); e) detailed descriptions of the sensing technique, material selection (e.g. its biocompatibility), and (f) power source and communication: power consumption, power source, and the type of machine or technique used to communicate with the sensor.

**Table 1:** Criteria per sensor characteristics for use in smart hip- and knee implants

Joint	Measuring range	Sensitivity	Resolution	Accuracy, precision	Durability	Size of sensor	Response time	Measuring speed	Measuring depth	Linearity, drift, hysteresis
Pressure and force sensors										
H	≥ 0-900N or 3 MPa [29]	≥ 1 N or 0.33 MPa [29]	≥ 1 N or 0.33 MPa [29]	≥ 1 N or 0.33 MPa [29]	≥ 20 years [9]	< 166 by 40 mm [40, 41]	≥ 0.1 s [30]	≤ 10 Hz [30]	≥ 76 mm [33]	L = 1^a^ [42] D&H = 0^b^
K	≥ 0-900N or 1.4 MPa [29]	≥ 1 N or 0.15 MPa [29]	≥ 1 N or 0.15 MPa [29]	≥ 1 N or 0.15 MPa [29]	≥ 15 years [10]	< 83 by 77 by 70 mm [43, 44]	≥ 0.1 s [30]	≤ 10 Hz [30]	≥ 10.2 mm [34]	L = 1^a^ [42] D&H = 0^b^
Strain sensors										
H	≥ 0-900N or 3 MPa [29]	≥ 1 N or 0.33 MPa [29]	≥ 1 N or 0.33 MPa [29]	≥ 1 N or 0.33 MPa [29]	≥ 20 years [9]	< 166 by 40 mm [40, 41]	≥ 0.1 s [30]	≤ 10 Hz [30]	≥ 76 mm [33]	L = 1^a^ [42] D&H = 0^b^
K	≥ 0-900N or ≤ 1.4 MPa [29]	≥ 1 N or 0.15 MPa [29]	≥ 1 N or 0.15 MPa [29]	≥ 1 N or 0.15 MPa [29]	≥ 15 years [10]	< 83 by 77 by 70 mm [43, 44]	≥ 0.1 s [30]	≤ 10 Hz [30]	≥ 10.2 mm [34]	L = 1^a^ [42] D&H = 0^b^
Acidity sensors										
H	≥ 6.7–7.4 decades [45]	≥ 0.1 decade [45]	≥ 0.1 decade [45]	≥ 0.1 decade [45]	≥ 20 years [9]	< 166 by 40 mm [40, 41]	≤ 1 day	≤ 1 measurement/day	≥ 76 mm [33]	L = 1^a^ [42] D&H = 0^b^
K	≥ 6.7–7.4 decades [45]	≥ 0.1 decade [45]	≥ 0.1 decade [45]	≥ 0.1 decade [45]	≥ 15 years [10]	< 83 by 77 by 70 mm [43, 44]	≤ 1 day	≤ 1 measurement/day	≥ 10.2 mm [34]	L = 1^a^ [42] D&H = 0^b^
Temperature sensors										
H	≥ 37 − 42 °C [46]	≥ 0.1 °C [46]	≥ 0.1 °C [46]	≥ 0.1 °C [46]	≥ 20 years [9]	< 166 by 40 mm [40, 41]	≤ 1 day	≤ 1 measurement/day	≥ 76 mm [33]	L = 1^a^ [42] D&H = 0^b^
K	≥ 37 − 42 °C [46]	≥ 0.1 °C [46]	≥ 0.1 °C [46]	≥ 0.1 °C [46]	≥ 15 years [10]	< 83 by 77 by 70 mm [43, 44]	≤ 1 day	≤ 1 measurement/day	≥ 10.2 mm [34]	L = 1^a^ [42] D&H = 0^b^
Bacteria sensors										
H	≤ 3.77 ppb [47]	any bacterial presence [48]	any bacterial presence [48]	any bacterial presence [48]	≥ 20 years [9]	< 166 by 40 mm [40, 41]	≤ 1 day	≤ 1 measurement/day	≥ 76 mm [33]	L = 1^a^ [42] D&H = 0^b^
K	≤ 3.77 ppb [47]	any bacterial presence [48]	any bacterial presence [48]	any bacterial presence [48]	≥ 15 years [10]	< 83 by 77 by 70 mm [43, 44]	≤ 1 day	≤ 1 measurement/day	≥ 10.2 mm [34]	L = 1^a^ [42] D&H = 0^b^

*H* hip, *K* knee

^a^Closest to 1

^b^Closest to 0

The data considering the sensor characteristics were analysed according to criteria derived from scientific literature and tailored to the specific characteristics of each sensor, as presented in Table 1. Satisfying these criteria suggests the sensor's applicability for detecting failure in hip and/or knee implants.

## Results

**Fig. 1:**
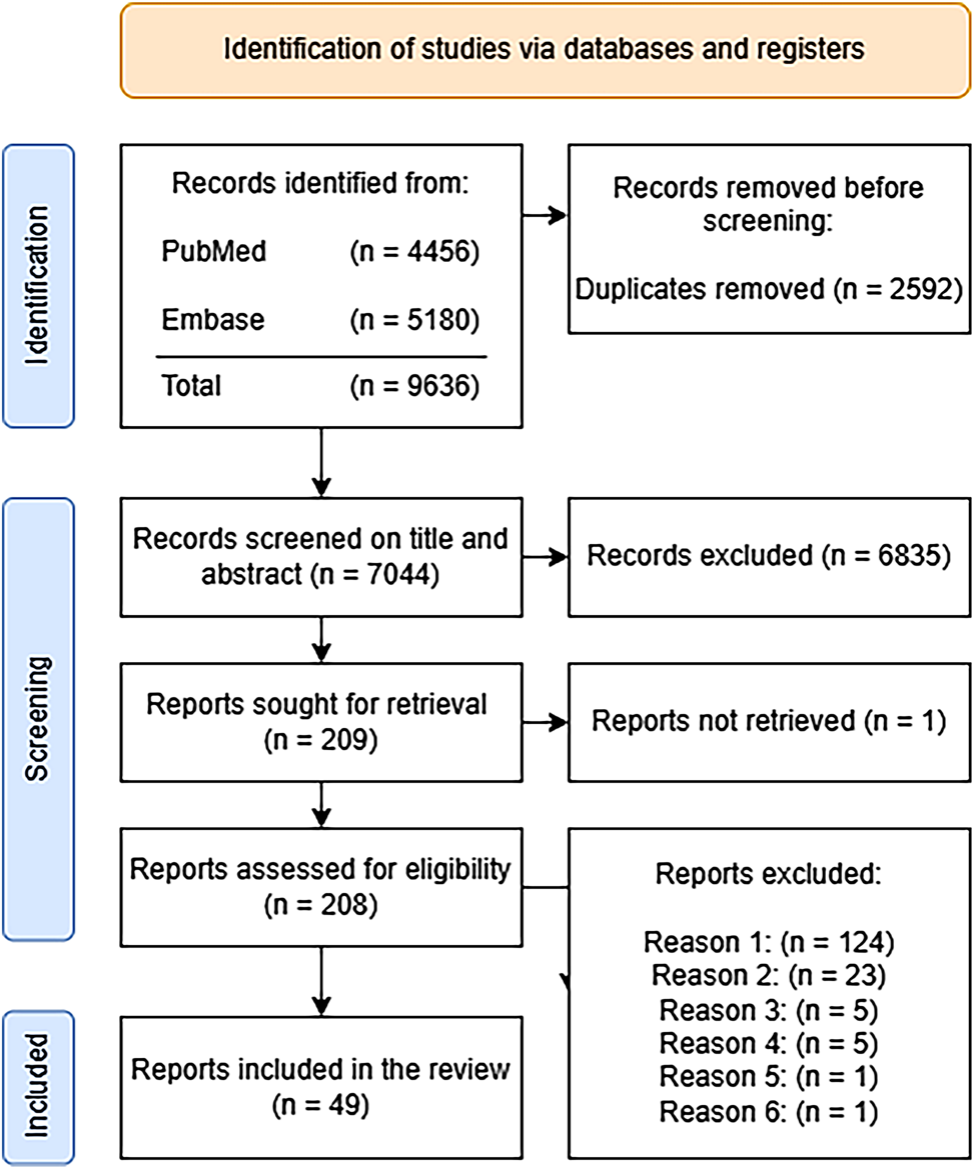
PRISMA 2020 flow diagram for new systematic reviews which included searches of the databases PubMed and Embase

The search resulted in 7044 articles. Based on the inclusion and exclusion criteria, two assessors selected 49 papers to be included. A flowchart of the screening process is given in Figure 1.

### Types of Sensors

The 49 included articles studied five different types of sensors (some studies included more than one type of sensor); 23 pressure and force sensors [49–71], six strain sensors [29, 30, 72–75], 15 acidity sensors [76–90], four temperature sensors [63, 72, 91, 92], and three bacteria sensors [93–95]. 43 of the sensors found were fully fabricated for their respective studies [29, 30, 49–55, 59–63, 65–70, 72–74, 76–84, 86–96] and six sensors contained parts that made up the sensing device were commercially available but modified to fit their own design (e.g. fully encapsulating the sensor or redesigning the pressure chamber) [56–58, 64, 71, 75].

Three of the sensors were investigated for integration in orthopaedic implants [29, 30, 95]. The remaining 46 sensors were designed for tracking the state of other implants (e.g. biofilm formation on a venous access port [93] and blockage pressure of a urethral stent [64]); monitoring the state of tissues (e.g. hypoxia in muscle tissue [88] and infection in wounds [74]); and unspecified biomedical applications. Table 2 provides an overview of the various applications with their testing environment.

**Table 2:** Applications of the different types of sensors

	Pressure and force	Strain	Acidity	Temperature	Bacteria
Testing environment					
In vitro	N = 22	N = 6	N = 13	N = 4	N = 3
In vivo	N = 13	N = 1	N = 4	N = 2	N = 0
Ex vivo	N = 1	N = 1	N = 3	N = 0	N = 0
Environment sensor was designed for					
Orthopaedic implant		[72, 73]			[98]
Implant surface			[92]		
Cerebrospinal	[49, 65]		[88]		
Intraocular	[49, 53, 55, 68, 71]				
Intravascular	[49, 51, 54, 56, 61, 62, 69, 70]	[76]			[96]
Transvaginal	[58]				
Urethral	[52, 64]				
Tumour site	[67]				
Nerve		[75]		[75]	
Wound		[77]	[81]		
Bone		[77]			
(Sub)cutaneous			[82, 91]		
Gastrointestinal tract	[59]		[84, 85, 92]		
Intramuscular			[89, 90]		
Organ surface			[82, 88]		
Artificial organ	[50]				
unspecified	[59, 60, 63, 67]		[78, 79, 81, 83, 86]	[63, 84, 93]	[97]

## Risk of Bias

**Fig. 2:**
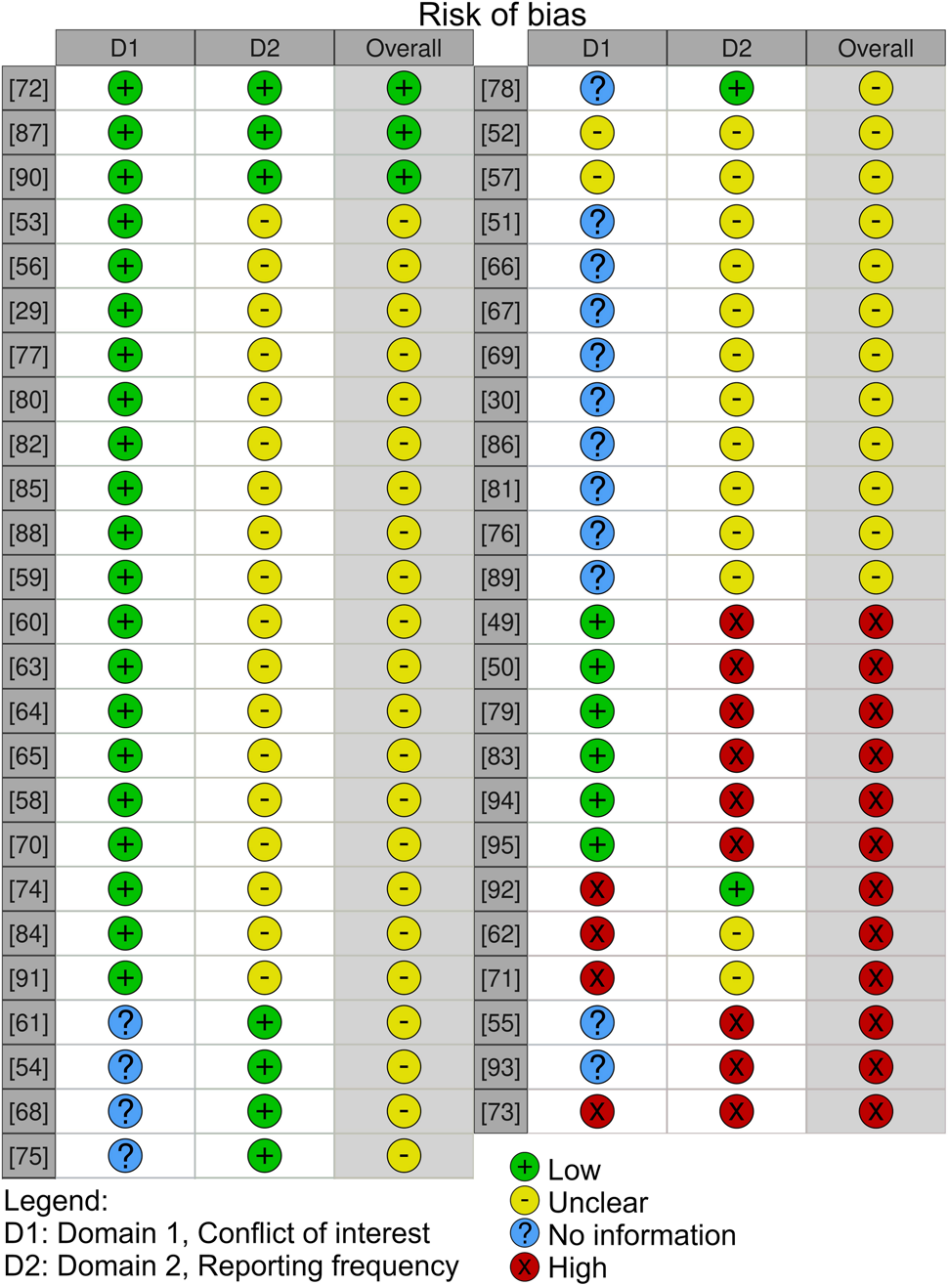
Risk of bias assessment for the included articles

Of the 49 articles, six authors declared a conflict of interest. Including one or more authors owning a patent related to the technology presented in the article [52, 62, 73, 92], one or more authors were connected to the source of financial aid that supported the research [57], and the research received funding of the corporate owners of the technology [71]. The risk of bias assessment is given in Figure 2.

### Mechanical (Force, Pressure and Strain) Sensors

Three types of most utilized mechanical sensors were identified: force and pressure sensors, and strain sensors. Table 3 presents detailed information about extracted data from the 29 included studies on mechanical sensors.

**Table 3:**
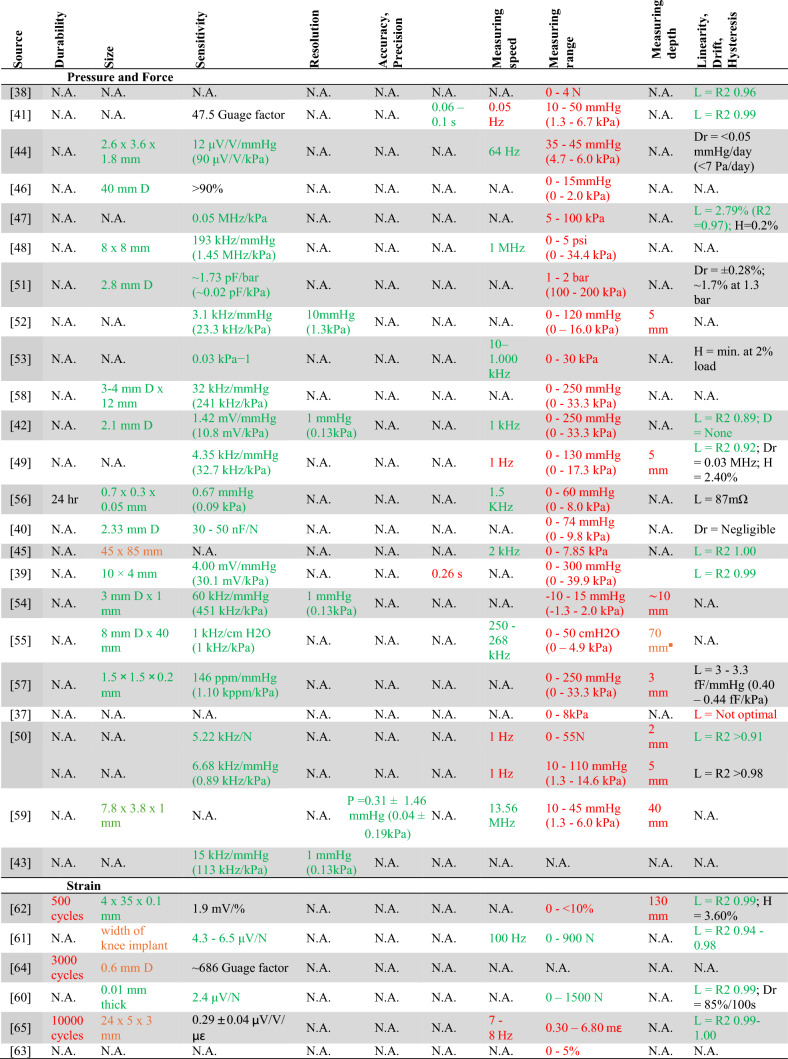
Specifications of all mechanical (pressure, force and strain) sensors

*N.A. * not applicable (the characteristic is not reported in the article), *D* diameter, *R *radius, *L *linearity, *A *accuracy, *P *precision, *Dr *drift, *H *hysteresis, *R2 *linear regression coefficient. *Red *not fitting criteria, *green *fitting criteria, *orange *only fitting criteria of knee implant

Of the 23 force and pressure sensors, the longest *in-vitro* test lasted three weeks for a sensor [54], the longest in vivo test lasted 30 weeks a pressure sensor [71]. Long-term testing beyond these tests was not reported. None of the pressure- and force sensors reported the *measuring range* necessary for use in the hip- and knee implant. One sensor does not report *measurement range*, but acceptable *sensitivity* (113 kHz/kPa) and *resolution* (0.13 kPa) [55].

Of the six strain sensors the longest test executed is 1000 cycles [73]. The longest (estimated) *durability* was 10.000 cycles [75]. Two strain sensors, both meant for measurements in the knee implant, have the sensor characteristics that encompasses the pressure presented during daily activities [29, 30]. One of them also fulfils the criteria for use in hip implants [30], the other sensor is too large and does not fit in or near a hip implant [29].

### Biochemical Sensors (Acidity, Bacteria, and Temperature Sensors)

The biochemical sensors include acidity, bacteria and temperature sensors. Table 4 presents detailed information about extracted data from the 20 included studies on biochemical sensors.

**Table 4:**
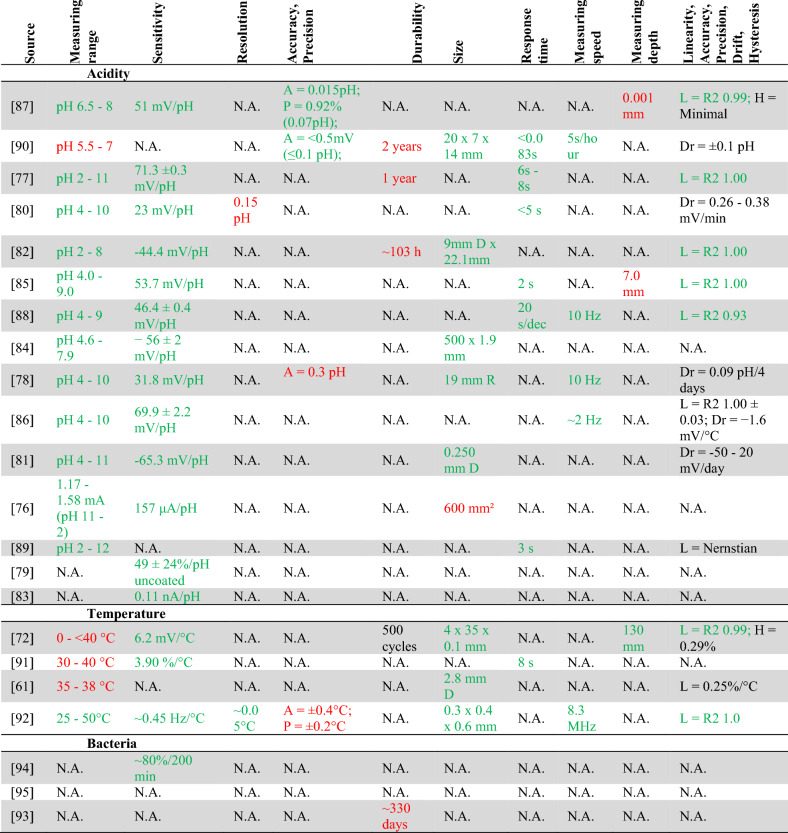
Specifications of all biochemical (acidity, temperature and bacteria) sensors

*N.A.* not applicable (the characteristic is not reported in the article), *D* diameter, *R* radius, *L* linearity, *Dr* drift, *H* hysteresis, *R2* linear regression coefficient. *Red* not fitting criteria, *green* fitting criteria, *orange* only fitting criteria of knee implant

Of the 13 articles on acidity sensors, the longest in vitro experiments lasted 18 months and the longest in vivo test lasted two hours (hypoxia sensor) [88]. The highest estimated *durability* is two years [90]. The sensor characteristics provided of seven sensors [79, 81, 83, 84, 86, 88, 89], fulfil the criteria. Two sensors have good characteristics, but a durability of 103 h [82] and 1 year [77].

For the four temperature sensors, the longest in vitro test lasted 48 hours [63], the most extended in vivo experiment ran for six weeks (invasive and inflammatory devices) [91] and the longest *durability* given as 500 cycles of 5% strain [72]. One sensor [72] has sufficient *measurement depth, sensitivity*, good *linearity*, and is within the *size* range*,* but it can only measure up to 40 °C. Another sensor [92] is within the acceptable *measurement* (25–50 °C) and *size* range and has good *measurement speed* and *resolution* but is not *accurate* enough (±0.4 °C) (*measure depth* is not provided).

For the three bacterial sensors, very limited information about the sensor characteristics was provided in these papers. The longest in vitro test lasted 20 hours [95] and the longest estimated *durability* is 330 days [93]. The only *sensitivity* reported, expressed as an 82.9% reduction in current after 200 minutes of culturing a biofilm [94].

## Discussion

This scoping review aims to provide an overview of current biomedical sensing solutions with potential for integration into hip and knee implants to measure periprosthetic infection, inflammation, implant loosening and wear. Sensors were identified for all four of these failure modes using pressure-, force-, strain-, acidity-, temperature-, and bacteria sensors. Two strain sensors and five acidity sensors meet all criteria required necessary for potential use in hip and knee implants. The two strain sensors were developed for knee implants [29, 30] and were both tested in a knee simulator, a machine that mimics the dynamic loads presented in the knee implant. For integration into the hip implant, strain variations due to its anatomical position in the hip should be studied using a similar hip simulator. Additionally, given the hip implant's size constraints, it should be explored whether one sensor could suffice instead of two. [62]. When disregarding the durability requirements relative to the lifespan of hip- and knee implants, two additional acidity sensors [77, 82] show potential. For translation to integration into the hip- and knee implant, the sensors must be further developed and tested e.g. investigate the effect of synovial fluid, and examine characteristics as the *linearity*, *accuracy*, *precision*, *drift*, and *hysteresis*.

None of the temperature sensors fulfil all criteria for use in a hip or knee implant. Vickers et al.'s review highlights the gap in current implant monitoring systems, particularly for detecting bacterial or pH-related infections/inflammations, which were found in this study. All three bacteria sensors could have potentially to be integrated in the hip- and knee implant [93–95]. However, their reporting frequency was only 4.8%, primarily due to the novelty of in vivo bacterial sensors. These sensors require further development and extensive testing before they can be integrated into hip and knee implants.

The durability criterium was not met for any sensors since long-term invasive sensing is a challenge for new sensor developments [97]. Encapsulating sensors within the implant, as shown by Damm et al. [22], could extend their lifespan by preventing biological occlusion. Parameters such as pressure, force, strain and temperature translate well through a layer of titanium, which makes these sensors still feasible in spite of their lower estimated durability.

### Risk of Bias

In this review, risk of bias is determined by reported conflicts of interest and reporting frequency. When authors have competing interests related to the sensor discussed, there is a higher chance of reporting bias, making the results less reliable. The more authors with conflicts of interest, the greater this risk becomes. This also applies to funding disclosures. When authors receive funding from parties with vested interests in positive sensor outcomes, the risk of reporting bias increases. Forty-three authors disclosed their funding sources, while six did not. One study was identified as having a high risk of bias [73], while four studies exhibited two domains with high risk and other domains rated as unclear or lacking sufficient information [55, 62, 66, 93]. Despite these limitations, all studies were included in this systematic review. The sensors in Tables 3 and 4 are ordered by reporting frequency (descending) and conflict of interest (ascending). This ordering places the most trustworthy sensors at the top of each category, making them priority candidates

Although the review provided a broad overview of sensors, many articles omitted key characteristics such as durability and size. The measuring range was the most commonly reported characteristic (n = 44), followed by sensitivity (n = 40). Articles with a lower reporting frequency are more likely to be considered as a viable option. For instance, all acidity sensors that gave an estimated durability are not sufficient for application in the hip- and knee implant, whereas the articles that did not report durability are still considered as an option.

### Strength and Limitations

This scoping review complements earlier reviews of sensorized hip and knee implants [5] by examining sensors currently used in other medical applications. It provides a comprehensive overview of biomedical sensors that could be integrated into instrumented hip and knee implants. By leveraging existing technologies rather than designing entirely new sensors, this approach could accelerate the development of smart implants.

Due to differences in measuring techniques between studies, various units are reported for similar sensor characteristics. For instance, the sensitivity of force and pressure sensors was expressed in multiple units: μV/mmHg, kHz/mmHg, μV/V/mmHg, mmHg, nF/N, kHz/N, Gauge factor, %, MHz/kPa, kPa − 1, pF/bar, pF/kPa, and kHz/cm H2O. Similar inconsistencies appear in sensor resolution measurements (mV/mmHg, mmHg, mbar L/s), strain sensor sensitivity (μV/N, mV/%, gauge factor, μV/V/με), and induction measurements presented in hertz (Hz) rather than voltage (V). Since many studies lacked sufficient information to convert units to a common standard, comparing results between studies proved challenging.

Regarding the study design of this review, several terms in the search string lacked corresponding MeSH terms in PubMed. While using these non-MeSH terms made the search less precise, it was necessary to ensure comprehensive coverage of all measured parameters. Additionally, PubMed and Embase databases were used to search for papers. Although Scopus might contain newer development articles, it was not included in the search. This decision was made because Embase significantly overlaps with Scopus, and Scopus's lack of subject headings and MeSH terms makes searches less precise—potentially missing relevant articles while returning irrelevant ones. A notable limitation of this study is the inclusion of six papers that exhibited high levels of bias. Specifically, one study featuring bacterial sensor [93] warrants further consideration for potential integration into a smart implant.

Short-term point-of-care measures remain essential but were not included in this review, since assays often employ single-use sensors, which necessitates frequent replacement or cleaning, limiting their practicality for continuous monitoring [98]. Biodegradable and bioabsorbable sensors were also excluded from this review due to their current limitations in terms of lifespan [99]. These exclusions could result in missing interesting sensors, as they could be further development to enhance their longevity.

### Recommendations for Follow-Up Research and Implications

Sensors initially designed for other applications must be modified and optimized for integration with hip or knee implants. Their adaptation for use across multiple anatomical sites, is essential to enhance their translational potential in orthopaedic implant monitoring. Building on the existing work by Damm et al., who have integrated pressure-, strain-, and force sensors into hip implants [22], there is an opportunity to further advance this technology. This requires research on developing sensors that can be mounted externally on implants and integrated into implant design. However, extensive testing and evaluation are necessary before implementation in patients. Overcoming these hurdles could pave the way for the widespread adoption of smart implants, potentially revolutionizing patient care and outcomes.

Given that patients between 46 and 50 years have 27.6% risk on revision surgery [6], a future research should prioritize developing sensors to detect wear particles, which play a crucial role in implant longevity. Wear particles are strongly linked to complications like aseptic loosening, periprosthetic osteolysis, and inflammatory reactions—all of which increase revision rates [23, 26, 27]. Current research in this area is limited, creating an urgent need for innovative approaches. Sensors that can precisely detect wear particles would advance our understanding of implant failure mechanisms and improve patient outcomes.

Early detection of implant failure also holds significant clinical implications. It could enable less invasive interventions, delaying revision surgeries and potentially extending implant lifespan [100]. Overcoming technical and logistical barriers will be essential to translating sensor technologies from (fundamental) research stage to higher technology readiness levels (TRL) and use in clinical practice. This includes further development of the sensors for this application (TRL 3 and 4) and rigorous testing in real-world conditions ensuring biocompatibility (TRL 5, 6 and 7), and developing robust data communication systems that can provide timely information to healthcare providers (TRL 6–9). By addressing these challenges, we can unlock the full potential of smart implant technology and create a brighter future for patients with hip replacements.

To conclude, our analysis identified two strain sensors that meet all criteria for hip and knee implants applications. Setting aside durability constraints relative implant lifespan, seven acidity sensors demonstrate potential. These sensors’ shorter-term functionality aligns well with the critical period for monitoring periprosthetic infections. We identified a significant gap in sensors capable of (early) detecting wear particles. Looking ahead, the sensors identified in this review should undergo further development and validation in vitro, in vivo, and ex vivo, to ensure their usability for integration into hip and/or knee implant. Future developments in continuous implant monitoring should prioritize (early) detection of wear particles, mitigating risks and extending sensor longevity.

## Appendix

### PubMed Search

("Biosensing Techniques"[Mesh] OR sensor[tiab] OR sensors[tiab] OR biosens*[tiab] OR"Potentiometry"[Mesh] OR"Ion-Selective Electrodes"[Mesh] OR ion selective electrod*[tiab] OR ion sensitive electrod*[tiab] OR"Electrochemical Techniques"[Mesh] OR electrochemical techni*[tiab])

AND

(Pressure senso*[tiab] OR strain gage*[tiab] OR strain senso*[tiab] OR strain gauge*[tiab] OR

"Thermometers"[Mesh] OR thermomete*[tiab] OR temperature senso*[tiab] OR"Prosthesis Failure"[Mesh] OR ((prosthe*[tiab] OR implan*[tiab]) AND (loos*[tiab] OR failure*[tiab] OR wear[tiab] OR malplacement*[tiab] OR malalign*[tiab])) OR"Hydrogen-Ion Concentration"[Mesh] OR hydrogen ion concentratio*[tiab] OR ph senso*[tiab] OR"Bacterial Infections"[Mesh] OR bacterial infectio*[tiab] OR bacterial diseas*[tiab] OR"Staphylococcus aureus"[Mesh] OR staphylococcus aureus[tiab] OR"Staphylococcus epidermidis"[Mesh] OR staphylococcus epidermidis[tiab] OR"C-Reactive Protein"[Mesh] OR c reactive protein[tiab] OR hscrp[tiab] OR hs crp[tiab] OR"Procalcitonin"[Mesh] OR calcitoni*[tiab] OR"L-Lactate Dehydrogenase"[Mesh] OR lactate dehydrogenase[tiab])

AND

("Reaction Time"[Mesh] OR reaction tim*[tiab] OR response tim*[tiab] OR response latenc*[tiab] OR response spee*[tiab] OR"Sensitivity and Specificity"[Mesh] OR specificity[tiab] OR sensitivity[tiab] OR"Limit of Detection"[Mesh] OR"Validation Study"[Publication Type] OR validation stud*[tiab] OR"Reproducibility of Results"[Mesh] OR reproducibility of findin*[tiab] OR reproducibility of resul*[tiab] OR reliability of resul*[tiab] OR result reliabilit*[tiab] OR validity of resul*[tiab] OR face validity[tiab] OR reliability and validity[tiab] OR validity and reliability[tiab] OR test retest reliabilit*[tiab] OR biocompatibility[tiab] OR biostability[tiab])

NOT

("SARS-CoV-2"[Mesh] OR sars cov 2*[tiab] OR sars coronavirus 2[tiab] OR severe acute respiratory syndrome coronavirus 2[tiab] OR 2019 novel coronaviru*[tiab] OR coronavirus disease 2019*[tiab] OR coronavirus disease 19*[tiab] OR 2019 ncov*[tiab] OR"covid-19"[Mesh] OR covid 19[tiab] OR covid19[tiab] OR wuhan seafood market pneumonia viru*[tiab] OR wuhan coronaviru*[tiab] OR

"Point-of-Care Testing"[Mesh] OR point of care[tiab] OR bedside testing[tiab] OR"Absorbable Implants"[Mesh] OR"Hydrogels"[Mesh] OR hydroge*[tiab] OR biodegradable*[tiab] OR bioabsorbable*[tiab] OR absorbable*[tiab] OR wearable[tiab] OR single use[tiab] OR"Food Industry"[Mesh] OR"Food"[Mesh] OR food[tiab] OR foods[tiab] OR"Immunoassay"[Mesh] OR immunoassa*[tiab] OR immunochromatographic assa*[tiab] OR"Prognosis"[Mesh] OR prognosis[tiab] OR prognostic facto*[tiab] OR"Environmental Monitoring"[Mesh] OR environmental monitoring[tiab] OR environmental surveillance[tiab] OR"Optical Devices"[Mesh] OR optical devic*[tiab] OR optical componen*[tiab] OR optical system*[tiab] OR Review[Publication Type] OR systematic review[Publication Type] OR meta-analysis[Publication Type])

AND

(2013:2023[pdat])

### Embase Search

(‘biosensing’/exp OR ‘sensor'/exp OR ‘ion selective electrode’/exp OR ‘electrochemical analysis'/exp OR ‘voltammetry’/exp OR (‘biosensing’ OR'sensor’ OR ‘sensors'OR ‘biosens*'OR ‘ion selective electrod*'OR ‘ion sensitive electrod*'OR ‘electrochemical techni*'OR ‘potentiometry'OR ‘voltammetry’):ab,ti,kw)

AND

(‘thermometer’/exp OR ‘prosthesis complication’/exp OR ‘pH’/exp OR ‘bacterial infection’/exp OR ‘Staphylococcus aureus’/exp OR ‘Staphylococcus epidermidis’/exp OR ‘C reactive protein’/exp OR ‘procalcitonin’/exp OR ‘lactate dehydrogenase’/exp OR (‘pressure senso*'OR ‘strain gage*'OR ‘strain senso*'OR ‘strain gauge*'OR ‘temperature senso*'OR ‘thermomete*'OR ((‘prosthe*'OR ‘implan*') AND (‘loos*'OR ‘failure*'OR ‘wear'OR ‘malplacement*'OR ‘malalign*')) OR ‘hydrogen ion concentratio*'OR ‘ph senso*'OR ‘bacterial infectio*'OR ‘bacterial diseas*'OR ‘staphylococcus aureus'OR ‘staphylococcus epidermidis'OR ‘c reactive protein'OR ‘hscrp'OR ‘hs crp’ OR ‘calcitoni*'OR ‘lactate dehydrogenase'):ab,ti,kw)

AND

(‘reaction time'/exp OR ‘sensitivity and specificity'/exp OR ‘limit of detection'/exp OR ‘validation study’/exp OR ‘reproducibility'/exp OR ‘biocompatibility'/exp OR ‘biostability'/exp OR (‘reaction tim*'OR ‘response tim*'OR ‘response latenc*'OR ‘response spee*'OR ‘specificity'OR ‘sensitivity'OR ‘validation stud*'OR ‘reproducibility of findin*'OR ‘reproducibility of resul*'OR ‘reliability of resul*'OR ‘result reliabilit*'OR ‘validity of resul*'OR ‘face validity'OR ‘reliability and validity'OR ‘validity and reliability'OR ‘test retest reliabilit*'OR ‘biocompatibility'OR ‘biostability'):ab,ti,kw)

NOT

(‘Severe acute respiratory syndrome coronavirus 2’/exp OR ‘coronavirus disease 2019’/exp OR ‘point of care testing’/exp OR ‘biodegradable implant'/exp OR ‘hydrogel’/exp OR ‘biodegradable'/exp OR ‘wearable computer’/exp OR ‘food industry'/exp OR ‘food'/exp OR ‘immunoassay’/exp OR ‘prognosis'/exp OR ‘environmental monitoring’/exp OR ‘optical instrumentation’/exp OR (‘sars cov 2*'OR ‘sars coronavirus 2'OR ‘severe acute respiratory syndrome coronavirus 2'OR ‘2019 novel coronaviru*'OR ‘coronavirus disease 2019*'OR ‘coronavirus disease 19*'OR ‘2019 ncov*'OR ‘covid 19'OR ‘covid19'OR ‘wuhan seafood market pneumonia viru*'OR ‘wuhan coronaviru*'OR ‘point of care'OR ‘bedside testing'OR ‘hydroge*'OR ‘biodegradable*'OR ‘bioabsorbable*'OR'absorbable*'OR ‘wearable'OR ‘single use'OR ‘food'OR ‘foods'OR ‘immunoassa*'OR ‘immunochromatographic assa*'OR ‘prognosis'OR ‘prognostic facto*'OR ‘environmental monitoring'OR ‘environmental surveillance'OR ‘optical devic*'OR ‘optical componen*'OR ‘optical system*'):ab,ti,kw)

### Materials, Power and Communication

#### Materials Used for Mechanical Sensors

For biocompatibility five of the force and pressure sensors and one strain sensor use a parylene coating [40, 45, 53, 57, 58, 64]. The other strain sensors use a polymer such as polyethylene and polyimide to accomplish the same [61–63, 65, 66]. The most often used material for creating a pressure sensing chamber is silicon. Sometimes it is used in combination with other materials such as platinum [50, 51] and nitrate [52], other times on its own [43, 46–48, 60]. Sensing area sizes such as these chambers, range from 1mm^2^ for a sensor integrated into a stent [58] to 2200mm^2^ for the intravaginal sensor [46].

#### Power and Communication Mechanical Sensors

15 mechanical sensors are passively powered [48–60, 63, 64]. These passive sensors do not require a power supply to be implanted as well. They are powered by using inductive (resonant) coupling to both power the sensor and to acquire data. In the inductively powered pressure sensors, copper coils were used as a data transferring system. The passive sensors are smaller in size compared to the actively powered sensors. Eight mechanical sensors are actively powered using piezo electrics, batteries, wires connected to a machine outside of the body, or the power supply of another implanted device [40, 43, 45, 46, 61, 62, 65, 66]. The highest power consumption, found among the active sensors, is 2.5 V [61, 62]. Among the passive pressure sensors, the most commonly used communication devices are an LC resonator and an induction coil. The other articles, including the strain and force sensors, mention varying communication devices of which five are physically connected to the sensor.

#### Materials Used for Biochemical Sensors

In four acidity sensors gold or platinum is used for the electrode [68, 69, 72, 79], and in four articles silver/silver chloride reference electrodes are used [73, 75, 79, 81]. To increase biocompatibility multiple materials are used to encase the sensors such as silicon [67], anti-fouling lipid layers [70], and parylene [71]. The effective sensing areas range from 200 to 300 nm wires for the gastroesophageal reflux monitoring sensor [74] to a 1 cm^2^ exposed area on an electrode made for unspecified biomedical applications [67]. In one of the temperature sensors, parylene is used to encapsulate the sensor to increase biocompatibility [83]. The other temperature sensor does not have the need for this since it is meant to be incapsulated by the medical device. The effective sensing areas ranging from 250 to 250 μm [83] to 10 to 30 mm [63] for the same two sensors. All bacterial sensors use completely different materials to create their sensors. For a sensor made for biofilm detection on medical devices, which has an effective sensing area size of 160 × 15 μm [85].

#### Power and Communication Biochemical Sensors

Of the articles focusing on measuring pH, two are passively powered [80, 81]. Of the other nine, seven do not mention a power source. The other two mention a power source that is used during testing, but not for the final product. Of the articles focusing on bacteria one is passively powered [86], and one uses a battery [84]. Of the articles focusing on temperature, one is passively powered using ultrasound waves [83]. The power consumption of the pH sensors ranges from 0 [75] to 2 × 1.55 V [73]. For the bacterial sensors the power consumption ranges from 2 [85]to 3 V [84]. For the temperature sensors power consumption is not mentioned. All articles on pH- and bacterial sensors that mention how data is extracted use a different communication device. The passive temperature sensors data extraction is done with an ultrasound imager [83].

("Biosensing Techniques"[Mesh] OR sensor[tiab] OR sensors[tiab] OR biosens*[tiab] OR

"Potentiometry"[Mesh] OR"Ion-Selective Electrodes"[Mesh] OR ion selective electrod*[tiab] OR ion sensitive electrod*[tiab] OR"Electrochemical Techniques"[Mesh] OR electrochemical techni*[tiab])
